# Hyperinfection with *Anisakis simplex sensu stricto*: observations from a cluster of two cases in France^☆^

**DOI:** 10.1016/j.fawpar.2025.e00282

**Published:** 2025-08-29

**Authors:** Manon Robert, Maureen Duflot, Fakhri Jeddi, Carelle Koudougou, Louise Durand, Florent Morio, Mélanie Gay, Patrice Le Pape, Rose-Anne Lavergne

**Affiliations:** a Parasitology and Mycology Laboratory, Nantes University Hospital, Nantes, France; b Anses, Laboratory for Food Safety, Boulogne-Sur-Mer, France; c Gastroenterology, Jules Verne Clinic, Nantes, France

**Keywords:** *Anisakis simplex s.s.*, Hyperinfection, Anisakidosis, Collective food poisoning, Raw fish consumption, Foodborne parasitic diseases

## Abstract

Anisakidosis is an infection resulting from the ingestion of raw or undercooked fish products infected by Anisakidae larvae. Most infections are self-limiting in humans. We report a case of a 70-year-old woman who developed gastrointestinal symptoms after consumption of home-made ceviche of hake. Forty larvae of Anisakidae were removed during digestive fibroscopy. Based on a molecular method the larvae were identified as *Anisakis simplex s. s.* A second family member who had eaten the same meal was also diagnosed with anisakidosis. This cluster of two cases underlines the need to raise awareness of this disease in the general population and highlights the importance of cooking fish or freezing it prior to consumption in raw homemade dishes.

## 1 Introduction

Anisakidosis is a zoonotic parasitic infection, caused by the larval stage of Anisakidae family. Final hosts of Anisakidae are marine mammals harbouring adults in their gastric chamber. Adults lay eggs that are eliminated in stool. These eggs evolve and release third-stage larvae (L3) in aquatic environments. L3 larvae are then ingested by an intermediate host, a zooplanctonic crustacean. Once an infected crustacean has been eaten by squid or fish ‐ intermediate paratenic host - the larvae migrate within the visceral cavity. Then the larvae become infective for marine mammals - the definitive host - through ingestion of fish/squid paratenic hosts (EFSA et al., 2024).

Accidental human infections may occur after ingestion of infected fishery products eaten raw, marinated or undercooked in absence of prior freezing (EFSA, 2010). Since humans are not natural hosts of Anisakidae, L3 are not able to develop further. However, the presence of Anisakidae larvae in the human body can induce allergic or gastrointestinal manifestations, which depend on the location of the larvae. During acute gastric anisakidosis, the repeated attempts of larvae to penetrate the gastric mucosa lead to intense epigastric pain that can be associated with nausea, vomiting, diarrhoea or urticaria. These symptoms typically occur within a couple of hours after the ingestion of a contaminated meal. In late intestinal forms, two to three days after ingestion of the larvae, patients experience severe abdominal pain sometimes with nausea, vomiting and diarrhoea (Adroher-Auroux and Benítez-Rodríguez, 2020). In humans, anisakidosis can be due to either *Anisakis*, *Pseudoterranova, Contracaecum*, or *Hysterothylacium* although formal identification using molecular tools is seldom performed (Shamsi and Barton, 2023). Anisakidosis is currently considered as an emerging zoonosis (McCarthy and Moore, 2000) and is the most common parasitic infection following the consumption of fishery products (EFSA, 2010). A recent literature review from 1965 to 2022 identified 762 cases of anisakidosis from 34 countries with a majority coming from Japan (Shamsi and Barton, 2023). The worldwide number of cases was estimated at around 76,000 cases up to 2017, although the true prevalence is unknown and likely underestimated (Bao et al., 2019; Shamsi and Barton, 2023). Here, we report two related cases of anisakiasis following the consumption of European hake within a single family, one involving hyperinfection, and discuss these observations in the context of recent data published on anisakidosis in Europe.

## 2 Case report

**Supplementary Fig. 1:**
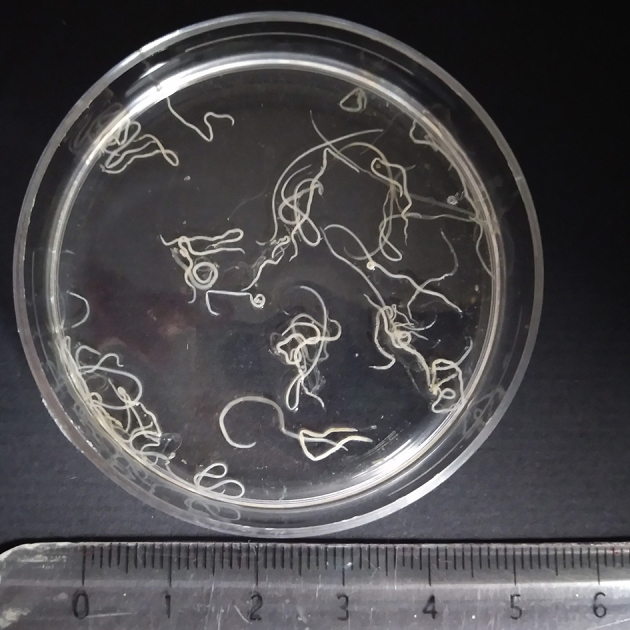
Photomicrograph of *Anisakis simplex sensu stricto* larvae removed from the gastric and colonic mucosa of a patient during esophagogastroduodenal fibroscopy and colonoscopy.

In the summer of 2021, a 70-year-old French woman underwent a scheduled esophagogastroduodenal fibroscopy and colonoscopy to investigate an iron deficiency anemia discovered two months earlier. On admission, she had been suffering from vomiting, epigastric and abdominal pain for 48 h. Symptoms started following the ingestion of homemade European hake ceviche (a dish with raw fish). During a 1.5 h-long digestive fibroscopy, multiple cylindrical worms attached to the gastric mucosa and to the colonic mucosa (mainly in the cecum and the ascending colon) were evidenced. About 40 white cylindrical worms 2 to 3.5 cm in length were finally removed (Supplementary Fig. 1). No white blood cell counts nor serological tests were carried out during the episode. The patient did not receive any antiparasitic treatment. The fish was bought at a local supermarket and prepared without pre-freezing. Of note, the patient reported seeing parasites in the pieces of fish when part of the meal had already been eaten by her and three other relatives, one of whom also experienced abdominal pain. Five larvae located in the gastric fundus were removed from this second patient during gastroscopy, which was performed five days after the consumption of the contaminated meal. No clinical data were available for the two other people who shared the same contaminated meal. Larvae of the index case were sent to the parasitology laboratory at Nantes University Hospital for identification. Stereomicroscopic examination of the worms confirmed the presence of L3 larvae of Anisakidae. Parasite DNA was isolated by manual extraction (NucleoSpin tissue, MachereyNagel) and the species was identified as *Anisakis simplex sensu stricto* using a previously published *Anisakis simplex s. s.*-specific real-time PCR (Paoletti et al., 2018). The importance of this observation lies in the hyperinfection of the index patient and the collective food poisoning due to *Anisakis simplex s. s.*

## 3 Discussion

Multiple infections with *Anisakis* larvae are not uncommon (Shimamura et al., 2016) but hyperinfection is unusual with only two similar cases reported in the literature in Europe. One hundred and forty larvae of *Anisakis simplex sensu lato* were removed from the stomach of a Portuguese woman (Baptista-Fernandes et al., 2017). Similarly, more than 200 larvae of *Anisakis simplex* were removed from the stomach of a Spanish woman (Jurado-Palomo et al., 2010). Most cases of anisakidosis currently described in the literature affect the stomach. Colonic anisakidosis is rare and as observed in our patient as well, larvae are most often found in the ascending colon and cecum (Mineta et al., 2006). Detailed anamnestic data, including food consumption in the days prior to the onset of symptoms, may allow anisakidosis to be suspected. In the cases described here, observation of the parasites in the ceviche led to early suspicion of Anisakidae infection, prior to gastroscopy. Diagnosis of gastric anisakidosis relies on upper endoscopy but intestinal anisakidosis is rather difficult to diagnose when larvae cannot be visualized by endoscopy. Eosinophilia is inconstant and of late onset, even in hyperinfected patients (Shimamura et al., 2016; Jurado-Palomo et al., 2010). Serological tests (IgG, IgE and IgA) are a cornerstone of the diagnosis of anisakidosis, particularly in case of chronic infection or allergic manifestations. Treatment of gastrointestinal anisakidosis relies primarily on physical removal of the larvae. Treatment with antiparasitic drugs (such as albendazole or ivermectin) has variable effectiveness (Cong and Elsheikha, 2021; Shimamura et al., 2016).

In Europe, it has been estimated that less than 20 cases of anisakidosis occur per year per country (Anses, 2017) and France is ranked third in terms of published cases, after Spain and Italy (Shamsi and Barton, 2023). A national retrospective survey of anisakidosis involving all University hospital parasitology laboratories in France between 2010 and 2014 reported a total of 37 cases, whatever the clinical presentation (gastrointestinal or allergic forms) (Yera et al., 2018), which was consistent with an earlier study in the same country which reported 21 cases from January 1985 to September 1987 (Hubert et al., 1989). However, the true prevalence of human anisakidosis in France is probably higher as not all cases can be expected to be referred to University hospitals. During the summer of 2018, 13 cases of anisakidosis were reported in the French province of Brittany following the consumption of raw hake or raw anchovies (communication to Mélanie Gay from Brittany Regional Health Agency and Finistère prefecture). In France, clustered cases of food poisoning - whatever the causative agent identified - must be notified to health authorities (Légifrance, 2023), and 1,200 to 1,800 cases have been reported annually since 2012 including no or few cases of anisakidosis (Santé Publique France, 2025). The completeness of the reporting of collective food poisoning caused by Anisakidae is unknown.

In humans, a vast majority of anisakidosis cases are due to *Anisakis simplex sensu lato*, and more specifically *A. simplex sensu stricto* and *A. pegreffii* (Adroher-Auroux and Benítez-Rodríguez, 2020). However, morphological distinction between theses sibling species (both belonging to the *A. simplex* complex) is impossible at the larval stage (Quiazon et al., 2008), but the species can be reliably identified by DNA sequencing and other molecular tools such as species-specific qPCR (Umehara et al., 2007). Species identification of the parasite in human cases helps to better understand the epidemiology of anisakidosis and to explore possible differences in pathology caused by different species.

More than 200 fish and cephalopod species have been described as paratenic hosts for Anisakidae larvae (Mattiucci et al., 2018). However, their importance in the fishing industry and in the circulation of these parasites is very variable. Indeed, prevalence may vary from 0 to 100 % depending on the species. Fish species including cod, salmon, hake, saithe, redfish, blue whiting, pouting, horse mackerel, sardine, anchovy, mackerel, and herring have been described with high infection levels (Adroher-Auroux and Benítez-Rodríguez, 2020). In France, a survey conducted in 2017 revealed a 70 % prevalence of Anisakidae infection in 37 samples of hake (fillets or whole fish) at the retail stage (Gay et al., 2012).

European regulations impose specific measures to reduce the risk of anisakidosis which include evisceration of fish as soon as possible after catching to prevent migration of larvae from the intestinal wall to the flesh. Furthermore, fish fillets must be visually inspected to ensure the absence of visible parasites. Finally, raw fish products must be obtained from fish that have undergone a sanitizing freezing treatment (for a minimum of 24 h at core at −20 °C or lower, or for a minimum of 15 h at core at −35 °C or lower). However, the present observations, where the fish had been purchased in a local supermarket, underline the importance of raising awareness about anisakidosis among the general population and remind of the importance of cooking fish to at least 60 °C for a minimum of 1 min at core or freezing fish before raw consumption during 7 days in a household freezer in case of homemade dishes (Anses, 2017), to avoid viable Anisakidae larvae and to reduce the risk of infection.

The following is the supplementary data related to this article.

## CRediT authorship contribution statement

**Manon Robert:** Writing – review & editing, Writing – original draft. **Maureen Duflot:** Writing – review & editing, Investigation, Conceptualization. **Fakhri Jeddi:** Writing – review & editing. **Carelle Koudougou:** Writing – review & editing, Resources. **Louise Durand:** Investigation. **Florent Morio:** Writing – review & editing. **Mélanie Gay:** Writing – review & editing, Conceptualization. **Patrice Le Pape:** Writing – review & editing. **Rose-Anne Lavergne:** Writing – review & editing, Supervision, Conceptualization.

## Funding source

None.

## Declaration of competing interest

The authors declare that they have no known competing financial interests or personal relationships that could have appeared to influence the work reported in this paper.
